# What does the nose know? Olfactory function predicts social network size in human

**DOI:** 10.1038/srep25026

**Published:** 2016-04-25

**Authors:** Lai-quan Zou, Zhuo-ya Yang, Yi Wang, Simon S. Y. Lui, An-tao Chen, Eric F. C. Cheung, Raymond C. K. Chan

**Affiliations:** 1 Neuropsychology and Applied Cognitive Neuroscience Laboratory, Key Laboratory of Mental Health, Institute of Psychology, Chinese Academy of Sciences, Beijing 100101, China; 2 University of Chinese Academy of Sciences, Beijing 100101, China; 3 Castle Peak Hospital, Hong Kong Special Administrative Region 999077, China; 4 School of Psychology, Southwest University, Chongqing 400715, China

## Abstract

Olfaction is an important medium of social communication in humans. However, it is not known whether olfactory function is associated with social network size. This study aimed to explore the underlying neural mechanism between olfactory function and social network. Thirty-one healthy individuals participated in this study. Social network size was estimated using the Social Network Index. Olfactory function was assessed with the Sniffin’ Stick Test. The results showed that there is a significant positive correlation between the size of an individual’s social network and their olfactory sensitivity. We also found that amygdala functional connectivity with the orbitofrontal cortex appeared to be related to olfactory sensitivity and social network size.

For most non-human mammals, smell is essential for recognizing social hierarchy and territory, and identifying conspecifics and predators^1^,^2^. As human developed language and other cognitive abilities for socialization, the olfactory genes for survival and social function under selection pressure decreased and pseudogenes accumulated^3^. However, olfaction is still an important medium of social communication in humans. In recent years, research has demonstrated that human odours can communicate information about gender, age, illness, kin recognition, mate choice, reproductive state, detection of danger and transient emotional states (e.g., sadness, stress, disgust)^4^,^5^. Clinical studies had found that individuals with psychiatric disorders (e.g., autism, schizophrenia, depression and so on) have poorer olfactory function^6^,^7^ and smaller social networks than the general population^8^,^9^,^10^. More specially, Dudova, *et al*.^11^ found that odour detection threshold, but not odour identification, was impaired in children with autism. Pollatos, *et al*.^12^ observed a significant negative correlation between olfactory sensitivity and depressive symptoms while olfactory discrimination was not related to depressive symptoms. In addition, extraversion had been associated with olfactory sensitivity^13^ and social network size^14^, defined as the total number of people that the participants had regular contact with. Based on the aforementioned findings, it is conceivable that olfactory sensitivity in human may be associated with social network size.

Recent studies have shown that healthy volunteers with larger social networks have more grey matter in brain regions implicated in adaptive social behaviours, such as the amygdala^15^,^16^,^17^, the subgenual anterior cingulate cortex (ACC)^15^, the ventromedial prefrontal cortex (vmPFC)^18^ and the orbitofrontal cortex (OFC)^17^,^19^. In addition, Bickart, *et al*.^20^ found that people with enhanced functional connectivity between the amygdala and specific regions such as the OFC, the vmPFC, the superior temporal sulcus (STS), the hippocampus and the fusiform gyrus have larger social network size. Ostensibly, these brain regions (i.e., the amygdala and the OFC) are also components of the olfactory system^21^. It is possible that a common neural circuitry subserves both olfaction and social network size, such that both social network size and olfactory sensitivity may be associated with amygdala-OFC functional connectivity.

In the present study, we aimed to test this hypothesis and examine 1) the association between olfactory sensitivity and social network size, and 2) the common neural basis between them. We hypothesized that there is a relationship between olfactory sensitivity and social network size, and both social network size and olfactory sensitivity are related to amygdala-OFC functional connectivity.

## Results

**Figure 1:**
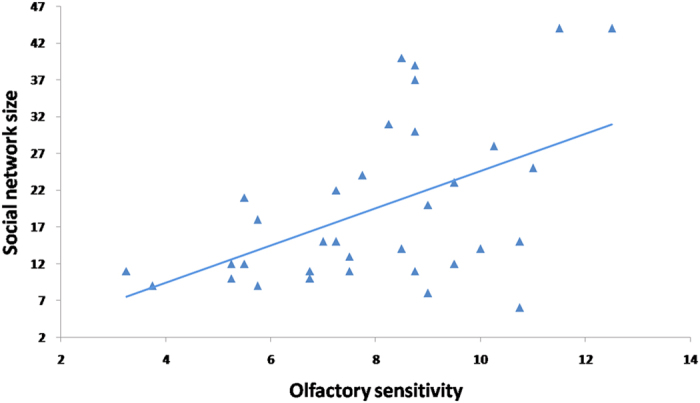
The relationship between olfactory threshold and social network size, after controlling for gender, age, education and smoking index (*r* = 0.51, *p* = 0.007).

Descriptive statistics of the demographic variables, social network size and olfactory performance are shown in Table 1. The behavioural results showed that social network size was positively correlated with olfactory threshold (*r* = 0.51, *p* = 0.004, power = 0.86) (see Fig. 1), even after controlling for gender, age, education level and smoking index (*r* = 0.51, *p* = 0.007, power = 0.86). However, social network size was not correlated with odour identification (*r* = 0.18, *p* = 0.32, power = 0.16) and discrimination (*r* = −0.11, *p* = 0.55, power = 0.09).

**Figure 2:**
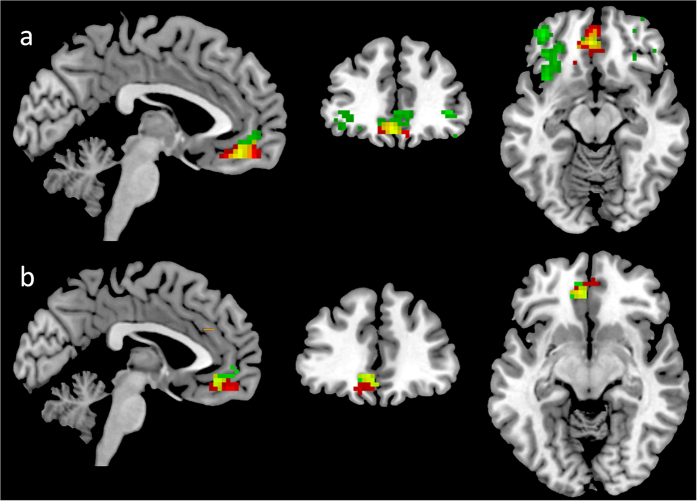
Amygdala-based functional connectivity that correlated with social network size and olfactory sensitivity. **(a)** Right amygdala-based functional connectivity. Red label: social network size; Green label: olfactory sensitivity; Yellow label: the overlap of function connectivity maps of social network size and olfactory sensitivity. **(b)** Left amygdala based functional connectivity. The colour labels are the same as (**a**). The threshold is set at *p* < 0.05, AlphaSim corrected.

Social network size predicted resting-state functional connectivity between the right amygdala and the left medial OFC ([−3, 50, −15], *r* = 0.60, see Fig. 2a). When the left amygdala was used as a seed, a positive correlation between amygdala–left medial OFC functional connectivity and social network size was observed ([0, 51, −15], *r* = 0.58, see Fig. 2b).

Right amygdala functional connectivity strength was correlated with olfactory threshold in the bilateral inferior OFC (Right: [42, 45, −2], *r* = 0.66; Left: [−39, 48, −12], *r* = 0.64) and the left medial OFC ([−3, 42, −15], *r* = 0.67) (see Fig. 2a). Olfactory threshold was positively and significantly associated with connectivity between the left amygdala and the left medial OFC ([−3, 39, −9], *r* = 0.50, see Fig. 2b).

Our results showed that both the right and the left amygdala functional connectivity of the social network size and olfactory threshold showed overlap in the left medial OFC (see Fig. 2).

## Discussion

The present study aimed to explore the correlation between olfactory sensitivity and social network size, and the common neural basis between them. To the best of our knowledge, this is the first study examining the relationship between olfactory function and social network size. We found a significant positive correlation between the size of an individual’s social network and their olfactory sensitivity. We extended this by showing that amygdala-OFC functional connectivity appeared to be related to both olfactory sensitivity and social network size.

Concerning the behavioural data, we did not find a significant correlation between social network size and odour identification or discrimination, while there was a correlation between social network size and olfactory sensitivity. The results are partially consistent with previous studies on individuals with social deficits. Dudova, *et al*.^11^ found that olfactory sensitivity, but not odour identification, is impaired in children with autism. In addition, Pollatos, *et al*.^12^ observed a significant negative correlation between olfactory sensitivity and depressive symptoms while olfactory discrimination was not related to depressive symptoms. One possible reason for this finding may be the fact that olfactory sensitivity test measured by the Sniffin’ Sticks test accesses information at the primary processing level, while the discrimination and identification tests access information at the secondary processing level. It is possible that olfactory sensitivity in the participants varied within the average range of “normal” olfactory sensitivity which is the necessary premise to assess olfactory identification and discrimination. However, olfactory identification and discrimination tests use odours at a clearly supra-threshold level, while olfactory sensitivity test uses odours at a much lower concentration.

We found a positive correlation between social network size and olfactory sensitivity. One possible explanation is that participants with higher olfactory sensitivity might be more sensitive to others’ body odour and could obtain more social chemical signals, which aid social communication. Several studies have also found that olfactory sensitivity may be related to social communication. Navarrete-Palacios, *et al*.^22^ found that olfactory threshold was associated with phases of the menstrual cycle in women and suggested that increased olfactory sensitivity during ovulation might facilitate mate finding^23^,^24^,^25^,^26^,^27^. Similarly, olfactory sensitivity and sexual desire were significantly correlated in normosmic young adult males^28^,^29^. In addition, people diagnosed with isolated congenital anosmia exhibit enhanced social insecurity, a reduced number of sexual relationships and increased risk of developing depressive symptoms^30^,^31^.

Furthermore, our results suggest that there is a common neural circuitry that subserves olfactory sensitivity and social network size. The amygdala functional connectivity associated with social network size and olfactory sensitivity showed overlap in the medial OFC, an orbitofrontal area that surrounds the olfactory sulcus. Previous studies have found that olfactory sensitivity was predicted by OFC volume or thickness^32^,^33^. In addition, we replicated the findings by Bickart, *et al*.^20^ linking social network size with amygdala-OFC connectivity. The amygdala and its interconnections with the PFC may play an important role in the interactions between emotion and cognition^34^. However, it should be noted that the fMRI data were collected at rest, not during performance of tasks involving smelling or social tasks that might activate the amygdala. So, while there may be correlations between amygdala functional connectivity with various brain regions and olfaction and social networks, it is not known whether the circuitry is common until one measures brain activity while actually performing the appropriate tasks. Future study should recruit an fMRI task involving a component to capture social network size.

Several limitations were inherent in the present study. First, the sample size was small, precluding any meaningful examination of gender difference. The post hoc power analysis is a concern, particularly for interpreting the negative results. Secondly, we did not control for the potential confounding effect of possible alteration in odour perception in female participants in relation to their menstrual cycle. Thirdly, although the Sniffin’ Sticks test and the Social Network Index have been tested in the Chinese population in previous studies, they have not been standardized for the Chinese population. In addition, previous studies have provided compelling support for olfactory dysfunction in several psychiatric disorders including schizophrenia^7^, Alzheimer’s disease and Parkinson’s disease^35^. As olfactory functioning and sociality, such as social network size, are closely linked, future research in olfaction may help in understanding the pathophysiological basis of psychiatric disorders.

In conclusion, the present study shows that there is an association between olfactory sensitivity and social network size in humans, and the strength of amygdala-OFC functional connectivity is related to olfactory sensitivity and social network size. Given the preliminary nature of the present findings, future study adopting a more rigorous research design and large sample size should be conducted to replicate and validate the association between olfactory sensitivity and social network size in humans.

## Materials and Methods

### Participants

Thirty-one healthy ethnic Chinese subjects (15 females, mean age = 29.13, *SD* = 8.03, range = 21–46 years; 16 males, mean age = 33.06, *SD* = 10.52, range = 20–50 years) participated in the present study. To be eligible for the study, a participant had to be a person who: 1) had lived in Chongqing, China for at least six months; 2) were right-handed; 3) were free from ear-nose-throat problem; 4) had no personal or family history of psychosis, depression, suicide, epilepsy or drug abuse. We also recorded the participants’ education level and smoking index (number of cigarettes smoked per day X length of smoking history in years). The research was approved by the ethics committee of the Institute of Psychology, Chinese Academy of Sciences and was carried out in accordance with the approved guidelines. Written informed consent was obtained from all the participants before the administration of the tests.

### Olfactory measures

Olfactory function was assessed birhinally using the standardized “Sniffin’ Sticks” test battery that included three tests, namely test for olfactory sensitivity/threshold, odour discrimination and odour identification^36^,^37^. Although the Sniffin’ Sticks test was standardized in European populations, it has been used extensively in Chinese populations in previous studies, such as Yang, *et al*.^38^, Yang, *et al*.^39^, Chen, *et al*.^40^, Gu and Li^41^, and Zou, *et al*.^42^. The results in these studies showed that the Sniffin’ Sticks olfactory test is suitable for application in Chinese populations.

The Sniffin’ Sticks test was performed according to the methods in Hummel, *et al*.^36^. For odour presentation, the experimenter removed the cap of the pens and placed the tip of the pen approximately 1–2 cm in front of both nostrils of the participants for approximately 3 s.

In the olfactory sensitivity test, odours were presented in 16 triplets of pens, two containing an almost odourless solvent and the other containing the odour (2-Phenylethanol) at a certain dilution (16 dilutions). The participants were asked to indicate the pen with the odourant. Presentation of triplets was separated by 20 s. Sensitivity was determined using a single-staircase, triple-forced choice procedure. Two successive correct identifications or one incorrect identification triggered a reversal of the staircase, i.e., the next higher or the next lower concentration step was presented, respectively. Seven reversals had to be obtained (including the starting point), and the sensitivity was defined as the mean of the last four staircase reversals.

In the odour discrimination test, 16 triplets of pens, with two containing the same odour and the third a different one, were presented to the participants. Participants were asked to identify which of the three pens smelled differently. Triplets were presented at intervals of approximately 20 s. The test result was the sum score of correctly identified pens.

In the odour identification test, 16 common odours had to be identified from a list of four descriptors (multiple forced-choice procedure). The interval between odour presentations was approximately 20 s. The test result was the sum score of correctly identified pens.

### Social network measures

Social network size was assessed by the Social Network Index^43^. It is a 12-item questionnaire measuring the number of people the participant saw or talked to in 12 regular types of social relationships (e.g., “How many close friends do you have? How many of these friends do you see or talk to at least once every two weeks?”). These included relationships with a spouse, parents, parents-in-law, children, other close family members, neighbours, friends, workmates, schoolmates, fellow volunteers, members of groups without religious affiliation, and religious groups. The social network size was the total number of people that the participants had regular contact with at least once every two weeks, computed by summing across the 12 social relationships.

### Behavioural analysis

Pearson correlation was conducted to calculate the associations between social network size and each of the olfactory tests (identification, discrimination and threshold/sensitivity). Since olfactory function may be affected by age, gender, education level and smoking index^7^, we also conducted partial correlation controlling for these variables. Post-hoc power analyses were performed using G*Power 3.1 software^44^ to determine whether the sample size could give acceptable results. Statistical power was computed as a function of significance level α, sample size, and effect size (i.e., correlation coefficient).

### MRI data acquisition

Resting state fMRI data were acquired on a 3T Siemens Trio scanner (Erlangen, Germany) at the Southwest University Imaging Centre. During the scan, participants were instructed to rest quietly with their eyes closed and not to fall asleep. We collected 200 contiguous EPI functional volumes (TR = 2500 ms; TE = 30 ms; flip angle = 90°, 40 slices, matrix = 64 × 64; FOV = 220 mm; voxel size = 3.4 × 3.4 × 3.5 mm). To aid spatial normalization and localization of functional data, a T1-weighted gradient rapid acquisition gradient echo (MPRAGE) sequence was used with the following parameters (TR = 2530 ms; TE = 2.34 ms; FOV = 256 mm; voxel size = 1 × 1 × 1 mm; matrix size = 256 × 256; flip angle = 7°, slice thickness = 1 mm).

### MRI data preprocessing

Image preprocessing was performed using Data Processing Assistant for Resting-State fMRI: Advanced Edition (DPARSFA, version 2.3, http://resting-fmri.sourceforge.net/) implemented in the MATLAB 2010a (Math Works, Natick, MA, USA) platform. The first 10 volumes of each participant were discarded for stabilization of the MR signal. The remaining 190 volumes were slice-time corrected and then re-aligned to the reference slice. Structural images were co-registered to individuals’ functional images, and segmented using the New Segment and DARTEL modules^45^. Head movement parameters were computed by estimating the translational movement in millimeters (*x, y, z*) and rotational motion in degrees (pitch, roll, yaw). Several nuisance variables (i.e. six motion parameters, white matter and cerebrospinal fluid signal) were regressed out from each voxel’s time series. Following this step, all images were normalized by DARTEL, and re-sampled to 3 mm isotropic voxels. Next, the images were spatially smoothed with an isotropic 4 mm full width at half maximum (FWHM) 3D-Gaussian kernel. Finally, a band-pass filter of 0.01 to 0.08 Hz was applied to the time series of each voxel to minimize the effect of low-frequency signal drift and high frequency variations.

### Functional connectivity analysis

Functional connectivity analysis was performed using a seed-region based approach^46^. The “seeds” were placed within the left and right amygdala as defined in the automated anatomical labeling-atlas (AAL)^47^. All subsequent analyses were conducted separately for each region of interest (the left or the right amygdala). For each participant, time series of the voxels within each ROI were averaged to generate reference time series, and then functional connectivity maps were produced by computing the correlation coefficients between the time series of the ROI and the other remaining voxels in the whole brain. These correlation maps were converted to *Z*-value maps using Fisher’s *r*-to-*z* transformation to improve the Gaussianity of their distribution. In order to generate group-level statistical maps showing how social network size and olfactory function modulate functional connectivity separately, a general linear model was constructed with olfactory performance or social network size as the predictor, and age, gender, education level and smoking index as covariates. For our hypothesis-driven focus on the function of the amygdala-OFC functional connectivity, the OFC mask, combined with the bilateral orbital superior frontal gyrus, the bilateral orbital middle frontal gyrus, the bilateral orbital inferior frontal gyrus and the bilateral medial orbital superior gyrus as defined in the AAL atlas, was used for group analysis. All analyses were corrected for multiple comparisons using the AlphaSim program (http://afni.nimh.nih.gov/pub/dist/doc/manual/AlphaSim.pdf) in the Resting-State fMRI Data Analysis Toolkit (REST) software package (http://resting-fmri.sourceforge.net). Statistical maps were created using a combined threshold of *p* < 0.05 and a minimum cluster size of 41 voxels, yielding a threshold of *p* < 0.05 AlphaSim corrected. The results were displayed using the MRIcron software (http://www.nitrc.org/projects/mricron).

## Additional Information

**How to cite this article**: Zou, L. *et al*. What does the nose know? Olfactory function predicts social network size in human. *Sci. Rep.* **6**, 25026; doi: 10.1038/srep25026 (2016).

## Figures and Tables

**Table 1:** Descriptive statistics of the demographic variables, social network size and olfactory performance.

	Minimum	Maximum	Mean	*SD*
Education (year)	4	21	13.23	3.55
Smoking index	0	42	4.55	9.72
Social network size	6	44	18.65	10.94
Identification	8	14	11.48	1.50
Discrimination	10	15	12.97	1.45
Threshold/Sensitivity	3.25	12.50	7.90	2.23
